# Feasibility and Safety of Washed Microbiota Transplantation as Salvage Therapy for Refractory Non‐CDI Gut Pathogens in Critically Ill Patients

**DOI:** 10.1111/1751-7915.70431

**Published:** 2026-08-13

**Authors:** Jie Xu, Aoli Diao, Rujun Ai, Xia Wu, Quan Wen, Yuyan Xiao, Xinyi He, You Yu, Zulun Zhang, Yeqing Wang, Zhihua Zhang, Faming Zhang, Bota Cui

**Affiliations:** ^1^ Department of Gastroenterology, Sir Run Run Hospital Nanjing Medical University Nanjing China; ^2^ Department of Microbiota Medicine and Medical Center for Digestive Diseases The Second Affiliated Hospital of Nanjing Medical University Nanjing China; ^3^ Key Lab of Holistic Integrative Enterology Nanjing Medical University Nanjing China; ^4^ Department of Gastroenterology, Punan Branch of Renji Hospital Shanghai Jiao Tong University School of Medicine (Punan Hospital in Pudong New District, Shanghai) Shanghai China; ^5^ Pediatric Intensive Care Unit, Beijing Children's Hospital Capital Medical University, National Center for Children's Health Beijing China; ^6^ Department of Digestive Children's Hospital of Nanjing Medical University Nanjing China

**Keywords:** faecal microbiota transplantation, gut infection, gut microbiota, pathogen decolonization, salvage therapy

## Abstract

Limited treatment options exist for refractory non‐*Clostridioides difficile* infection (non‐CDI) of the gut in critically ill patients; whether washed microbiota transplantation (WMT) is feasible and safe in this heterogeneous population is unknown. We retrospectively analysed prospectively registered consecutive cases (NCT03895593) between September 2015 and August 2022, in which severe non‐CDI gut pathogens were identified, and rescue WMT was administered after standard treatment failure. Primary outcomes were the clinical outcome and adverse events graded by CTCAE v5.0. Secondary descriptive outcomes were the change in total abdominal symptom score (TASS) on day 7, microbiological clearance, and 12‐week survival. Ten patients underwent 29 WMT infusions. Fifty percentage of patients (5/10) obtained clinical cure after WMT. Twenty percentage of patients (2/10) achieved clinical improvement and 30% (3/10) showed no response 7 days after WMT. One transient WMT‐related fever occurred (1/29, 3.45%). No serious WMT‐attributed events were observed. TASS decreased significantly (*p* = 0.007). Microbiological clearance was documented in 3 of 4 patients with available post‐WMT stool bacteriological test results. This case series provides preliminary evidence supporting the feasibility and potential efficacy of WMT for refractory non‐CDI gut pathogens.

## Graphical abstract

Ten patients with definite gut infection evidence beyond CDI underwent 29 WMT infusions. 50% (5/10) of patients obtained clinical cure after WMT. 20% (2/10) of patients achieved clinical improvement and 30% (3/10) showed no response 7 days after WMT. One transient WMT‐related fever occurred (1/29, 3.45%).

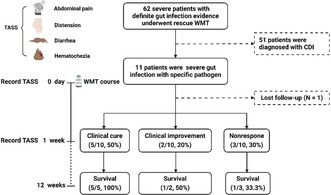

## 1 Introduction

Gut infections among severely ill patients, particularly in intensive care unit (ICU) patients, represent a significant cause of morbidity and mortality (Cohen et al. 2017; Enosi Tuipulotu et al. 2021; Hu et al. 2021; Schwartz et al. 2023). The high recurrence rate, increased healthcare consumption, and limited treatment options have created a huge burden for both society and patients. Moreover, refractory intestinal infections tend to appear in severe patients with multiple comorbidities or immunosuppression status (Qin et al. 2020; Cho et al. 2024; Park et al. 2024). Such patients rarely benefit from conventional antibiotic therapy and may worsen due to multidrug‐resistant organisms (MDROs) and gut microbiota dysbiosis caused by antibiotics (Dai et al. 2019; Maier et al. 2021). Studies have shown that in critically ill patients, early treatment with anti‐anaerobic antibiotics—which are recommended by the Infectious Diseases Society of America (IDSA) guidelines for treating anaerobic pathogens—increases the risk of adverse clinical outcomes (Chanderraj et al. 2023). It is, therefore, imperative to develop a treatment strategy for gastrointestinal (GI) pathogen infections in severely ill patients.

The human gut microbiota plays a crucial role in the maintenance of health. Recent studies have shown that alterations in the gut microbiota are closely associated with susceptibility to infections, colonization by pathogenic microbiota, and mortality (Shah et al. 2021; Garcia et al. 2022; Park et al. 2024). Pathogenic microbiota can cause disturbances to the gut microbiota, thereby exacerbating the severity of infections (Kienesberger et al. 2022; Lesniak et al. 2022; Le Guern et al. 2023). Furthermore, such disturbances can increase the likelihood of developing sepsis and organ dysfunction in critically ill patients (Adelman et al. 2020). Consequently, microbiome‐based interventions, such as faecal microbiota transplantation (FMT), may offer a potential therapeutic approach for severe gut infections. Previous studies on FMT have predominantly focused on the treatment of *Clostridioides difficile* infection (CDI) (Van Prehn et al. 2021; Wu et al. 2023; Nooij et al. 2024; Yadegar et al. 2024). However, studies investigating FMT for severe bacterial gut infections beyond CDI remains limited. Promising results from existing studies indicate that 24 of 35 (68.6%) patients achieved decolonization of MDROs within 1 year after FMT (Seong et al. 2020), indicating the therapeutic value of FMT for severe gut infections caused by pathogenic microbiota beyond CDI (Lahtinen et al. 2017; Huttner et al. 2019; Torres Soto et al. 2019; Ghani et al. 2021).

Washed microbiota transplantation (WMT) is a next generation of FMT (Zhang et al. 2026), based on the automatic microfiltration machine, repeated washing process, and delivery routes (Zhang, Lu, et al. 2020). WMT significantly decreased the incidence of adverse events. Studies have shown that the incidence of fever decreased from 19.4% (6/31) in manual FMT to 2.7% (24/902) in WMT in patients with ulcerative colitis (UC) (Lu et al. 2022). However, there is still no evidence for WMT in severe patients with gut infections caused by identified pathogens beyond CDI. Thus, we aim to evaluate the efficacy and safety of WMT therapy in severe patients infected with refractory non‐CDI gut pathogens.

## 2 Methods

### 2.1 Study Design and Patients

This was a prospective, observational, and multiple‐centre study conducted at the Second Affiliated Hospital of Nanjing Medical University (NJMU) and seven other medical centres applying for rescue WMT through the China Microbiota Transplantation System (CMTS) from September 2015 to August 2022. This is a part of the registered study of the long‐term safety and efficacy of rescue FMT for refractory intestinal infections (ClinicalTrial.gov, Number NCT03895593). The study included patients with gut infections who applied for rescue FMT with definite evidence of intestinal pathogenicity. Exclusion criteria included insufficient evidence of intestinal pathogenic infection, presented symptoms resulting from CDI, or follow‐up of less than 7 days. The CMTS provided the rescue WMT therapy course, and approval was granted by the institutional ethical review committee at the Second Affiliated Hospital of NJMU. Written informed consent was obtained from all patients or legal representatives of the patients. The ages, diagnosis, use of antibiotics, duration of the infection, the total abdominal symptom score (TASS) (Wu et al. 2023) at baseline and 7 days after WMT, and the outcomes of pathogenic evidence after WMT were collected.

### 2.2 WMT Procedure

The donors were screened using strict criteria in accordance with the consensus methodology for WMT (Zhang et al. 2026). The duration from faeces donation to the delivery to patients' gut or cryopreservation (−80°C) is strictly limited within 1 h (Zhang et al. 2026). The frozen washed microbiota suspension was transported cryogenically to other medical centres. WMT could be delivered through a gastric tube, nasojejunal tube, gastroendoscopy, colonic transendoscopic enteral tube (TET), enema, and colonoscopy (Zhang et al. 2026).

### 2.3 Definition of the Outcomes

Clinical cure was defined as TASS equal to zero at 7 days post‐WMT, or evidence of gut infection turning negative with TASS less than or equal to one point at 7 days post‐WMT. Clinical improvement was defined as the TASS decreasing by more than three points or decreasing to less than or equal to one point at 7 days post‐WMT. WMT nonresponse was defined as no significant improvement or even worsening of abdominal symptoms at 7 days post‐WMT. Additionally, we collected information on adverse events and general clinical outcomes of patients at the 12‐week follow‐up as exploratory outcomes.

### 2.4 Statistical Analysis

Analyses were performed using SPSS (Chicago, IL, USA). Changes of TASS and defecation scores were analysed by Paired Samples Wilcoxon Signed Rank Test. A 2‐tailed *p* < 0.05 was considered statistically significant.

## 3 Results

### 3.1 Study Population‐Baseline

**FIGURE 1:**
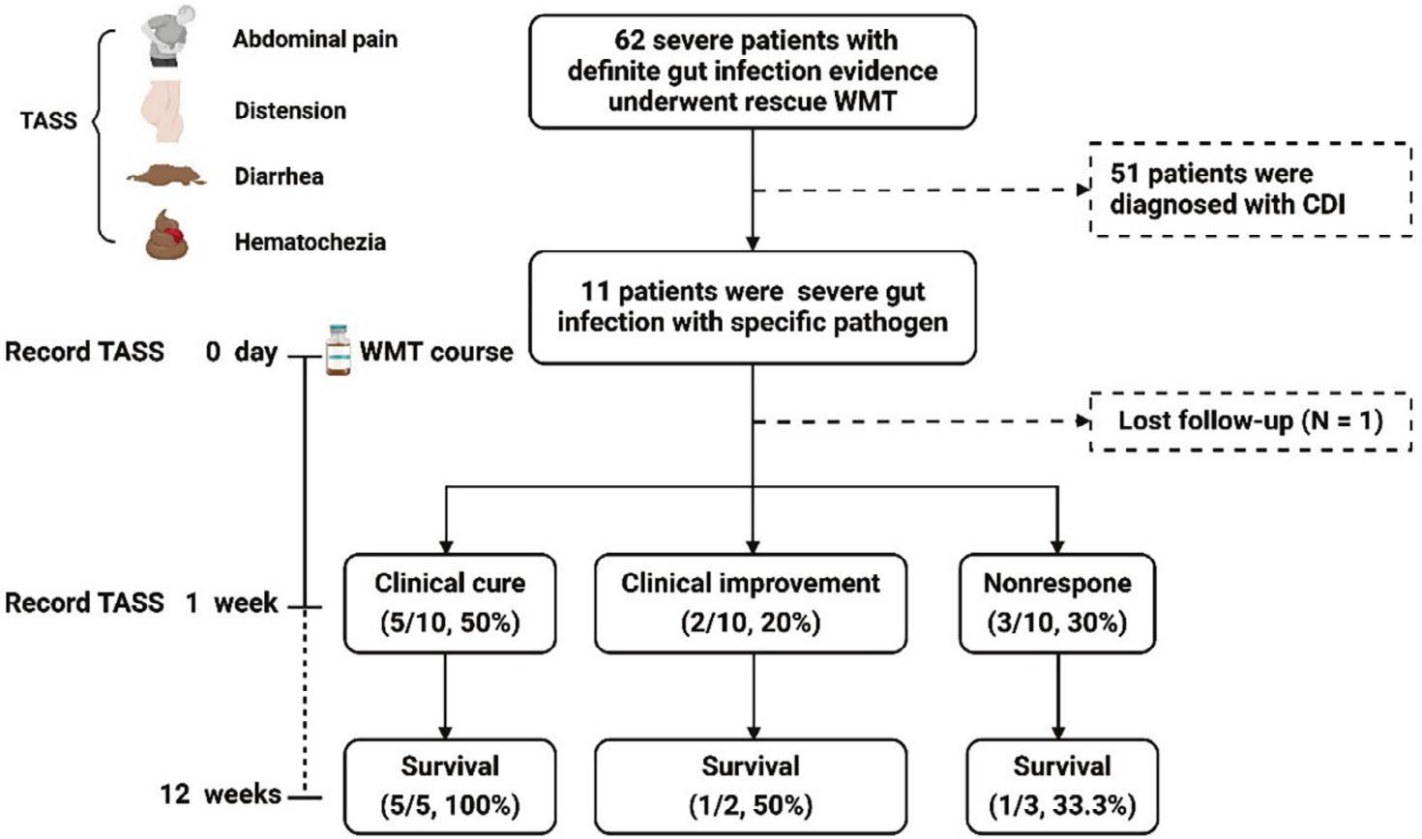
Flowchart of the study. WMT, washed microbiota transplantation; TASS, total abdominal symptom score, total scores of four components: abdominal pain, distension, defecation, and hematochezia. *Definite gut infection evidence includes clinical symptoms caused by pathogenic gut infection and positive pathogenic evidence. Positive pathogenic evidence contains stool culture, anal mucosal swab culture, or polymerase chain reaction (PCR). Patients (*n* = 10) who have completed the 7‐day after WMT follow‐up were divided into clinical cure, clinical improvement, and nonresponse according to their TASS accompanied with pathogenic evidence. **Survival rates were further collected in each group of clinical outcomes.

This study included 11 patients with definite gut infection evidence beyond CDI who underwent rescue WMT. All involved patients have been diagnosed with intestinal infections based on pathogenic evidence and clinical symptoms before the rescue. One patient lost our follow‐up and was excluded from the analysis Figure 1. Characteristics of the analysed ten patients were detailed in Table 1. The ages of the patients ranged from 4 months to 85 years old, with four patients less than 14 years old.

**TABLE 1:** Characteristic of 10 patients.

Pt	Age (month or year)	Microbiological Pathogen (stool or anal swab sample)	Primary diagnosis at the time of rescue	Condition of extra‐intestine infection	TASS before WMT	TASS after WMT	WMT‐related adverse event	Efficacy
Pt1	37 year	*Candida glabrata*	Post‐HSCT; GVHD; hemorrhagic cystitis	RTI; BSI	6	1	None	Clinical improvement
Pt2	11 year	*Klebsiella pneumoniae*	Crohn's disease; miliary tuberculosis; pancreatitis	RTI	4	0	None	Clinical cure
Pt3	11 year	*Salmonella typhimurium*	Crohn's disease	—	5	0	None	Clinical cure
Pt4	4 month	*Clostridium botulinum type B*	Severe botulism, respiratory failure, aspiration pneumonia; myocardial injury	RTI	0	0	Fever^a^	Clinical cure
Pt5	6 year	*Pseudomonas aeruginosa*	Post‐HSCT; Shock; mitochondrial myopathy; gastrointestinal haemorrhage	RTI; BSI	12	10	None	Nonresponse
Pt6	41 year	*Klebsiella pneumoniae*	Aortic dissection; gastrointestinal haemorrhage; shock; acute kidney injury	RTI; SSTI; UTI	3	1	None	Clinical improvement
Pt7	65 year	*Klebsiella oxytoca*	AIHA; gastrointestinal haemorrhagecon	RTI; SSTI	5	0	None	Clinical cure
Pt8	85 year	*Enterococcus*	*Cryptococcus neoformans* meningitis; cholecystitis; hepatic impairment; respiratory tract infections	RTI; BSI; UTI	5	4	None	Nonresponse
Pt9	73 year	*Klebsiella pneumoniae*	Severe pneumonia; intracerebral haemorrhage; diabetes mellitus; hypertension	RTI; BSI	4	2	None	Nonresponse
Pt10	52 year	*Candida albicans*	Pheochromocytoma; cardiac arrest	RTI; SSTI	3	0	None	Clinical cure

Abbreviations: AIHA, autoimmune haemolytic anaemia; BSI, bloodstream infection; GVHD, graft versus host disease; post‐HSCT, post Haematopoietic stem cell transplantation; Pt, patient; RTI, respiratory tract infection; SSTI, skin and soft tissue infection; TASS, total abdominal symptom score; UTI, urinary tract infection.

^a^ Pt4 had an AE of fever, which was assessed as probably WMT‐related. However, the fever relieved itself without medical treatment.

**FIGURE 2:**
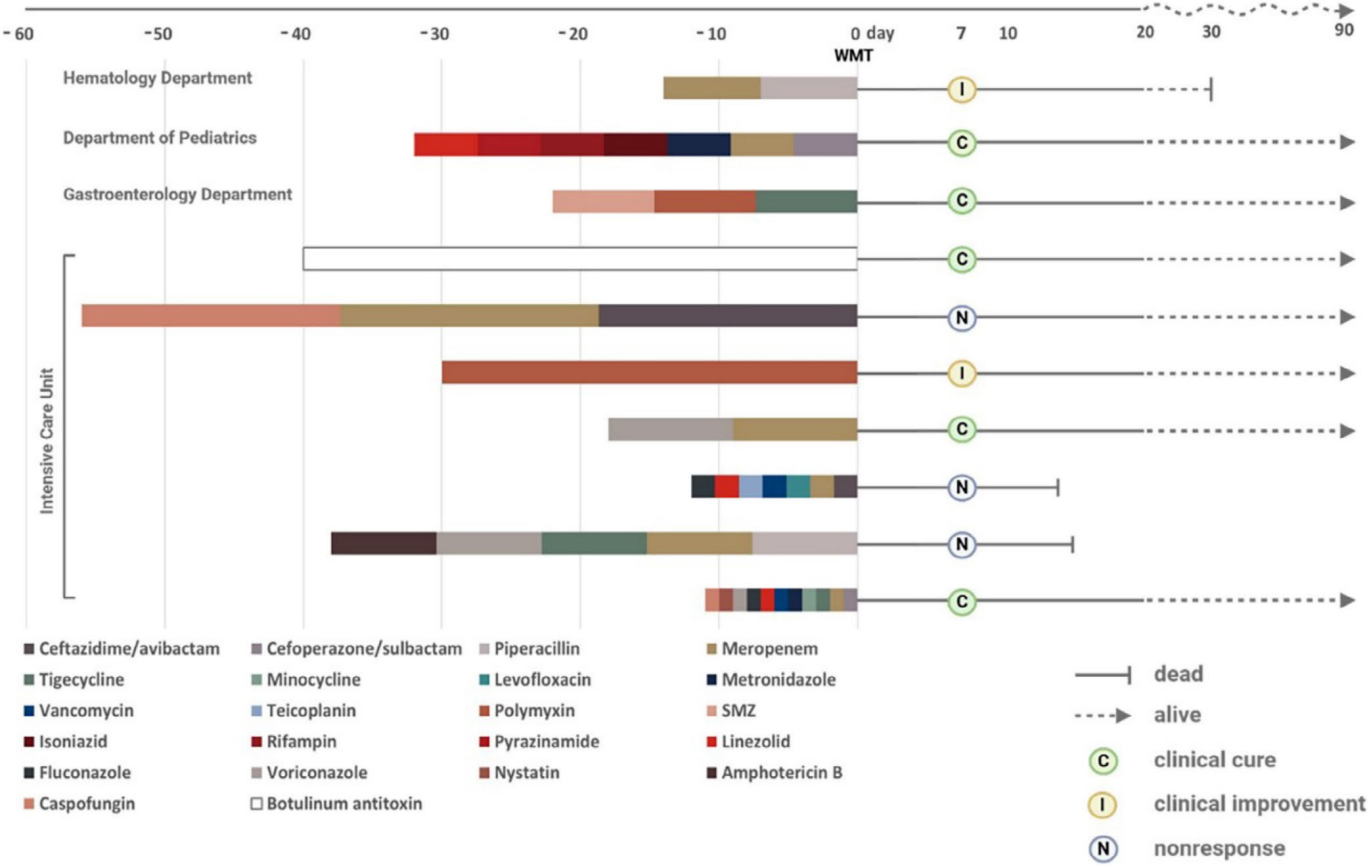
Conditions of patients before and after WMT rescue therapy. The sources of patients who underwent WMT rescue are illustrated at the left of the bars. The disease durations (days) of infection before WMT rescue are expressed with the length of the bars. The types of antibiotics used before WMT rescue is represented in bars of different colours. The clinical outcomes of patients after WMT are shown at the right of the bars.

As shown in Table 1 and Figure 2, these patients had complex combinations before undergoing WMT rescue therapy. Four patients had pneumonia. Three patients were diagnosed with gastrointestinal bleeding. Two patients were in post‐HSCT status. Two patients had respiratory failure. Two patients were diagnosed with shock. One patient had multiple organ failure (MOF). The TASS of Patient 4 before WMT was zero. This infant (Patient 4) had typical botulism symptoms with a positive result for *Clostridium botulinum type B*. Moreover, repeated stool sample polymerase chain reaction (PCR) testing remained positive for *C. botulinum type B* during 1‐month botulinum antitoxin treatment. Seven of ten received medical care in the ICU at the time of rescue, with the other three patients from the department of paediatrics, gastroenterology, and haematology, respectively Figure 2. The median time of the course of gut infection before seeking WMT was 26 days (range 11–56 days) Figure 2. All the patients were confirmed with evidence, and the pathogenic information is illustrated in Table 1. The types of antibiotics used during this course before WMT were demonstrated in Figure 2.

Nine of ten (90%) patients had complicated extra‐intestinal infections, including 90% (9/10) with respiratory tract infection, 40% (4/10) with bloodstream infection, 30% (3/10) with skin and soft tissue infection, and 20% (2/10) with urinary tract infection, as shown in Table 1.

### 3.2 WMT Delivery Routes and Courses

The delivery routes are illustrated in Table S1. Six patients underwent the WMT through mid‐gut, two patients through colonic TET, and another two patients through enema. The course of WMT was defined as undergoing consecutive WMT treatments under one clinical decision‐making. All of the ten patients accepted one course of WMT. The frequency of the WMT course depended on delivery routes, patient tolerance and disease severity. Although Patient 5 achieved nonresponse after the WMT treatment, a marginal symptomatic improvement was observed. However, Patient 5 emerged with a clue of recurrent infection during our exploratory observation. Thus, this patient applied the second WMT rescue according to the following clinical decision‐making.

### 3.3 Clinical Outcomes of WMT

**FIGURE 3:**
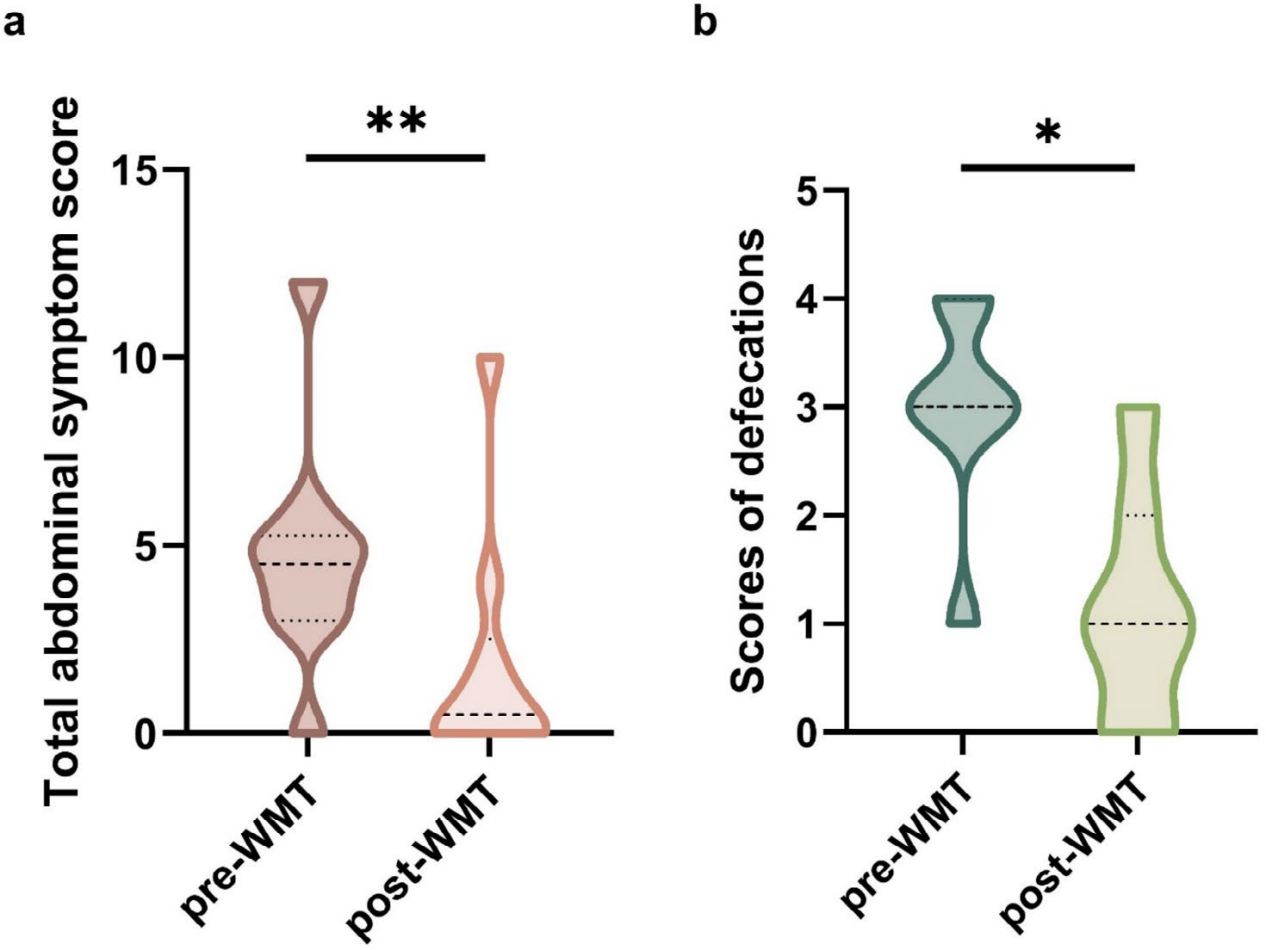
Efficacy of WMT rescue. (a) the statistical comparison of TASS between pre‐WMT and post‐WMT (*p* = 0.007); (b) the scores of defecations (*p* = 0.039) between pre‐WMT and post‐WMT. *p < 0.05, **p < 0.01.

TASS was significantly reduced (Wilcoxon signed rank test, *p* = 0.007) 7 days after WMT Figure 3a. Besides, patients with increased scores of defecations (*n* = 7) declined significantly (Wilcoxon signed rank test, *p* = 0.039) Figure 3b. As presented in Table 1, five patients obtained clinical cure, among which all five patients' abdominal symptoms disappeared after WMT, and three patients' intestinal pathogen evidence turned negative after WMT. Two patients achieved clinical improvement and three patients turned out to be nonresponse 7 days after WMT.

### 3.4 Exploratory Outcome

We continued to follow these patients until 12 weeks as an exploratory observation. The survival rates in the clinical cure group, clinical improvement group, and nonresponse group at 12 weeks are 100% (5/5), 50% (1/2), and 33.3% (1/3), respectively. Three of ten (30%) patients died within the 12‐week exploratory observation. Three patients' deaths were not related to WMT: Patient 1 died within 1 month, but the clinical symptoms of defecation and abdominal pain were improved, which was the main desire of the patient's application for WMT rescue. This patient had been diagnosed with GVHD with a bad situation before rescue WMT, and he died of multiple organ failure (MOF) in the end. Patient 8 died of sepsis, which was confirmed in the ICU before undergoing WMT. Patient 9 obtained an improvement in laboratory examination of serum infection factors and stool samples after WMT. However, this patient with multiple complications had heart failure, and it finally deprived him of life.

### 3.5 Adverse Events

One adverse event (1/29, 3.45%) occurred within 7 days of follow‐up. Patient 4 had transient FMT‐related AEs during follow‐up; no serious AE occurred in this research. This patient developed a fever after the WMT intervention, which was considered probably related. The fever was self‐limiting and relieved without any medical intervention Table 1.

## 4 Discussion

The data from this small‐sample study tentatively suggest that WMT may serve as a potential treatment option for severely ill patients with refractory gut infections caused by identified pathogens beyond CDI. Patients with comorbidities are commonly excluded from most clinical research due to their complex, multifactorial complications and challenging therapeutic management, which has resulted in a paucity of evidence to guide treatment in this population. Notably, Patient 4—who presented with a *C. botulinum type B* infection accompanied by severe neurological symptoms but no abdominal manifestations, and who did not benefit from botulinum antitoxin—achieved clinical cure. This observation preliminarily suggests that WMT may have potential value in pathogen decolonization. Collectively, these preliminary findings offer a potential treatment strategy for severe patients with refractory infections and comorbidities, particularly in the intensive care unit setting. Furthermore, this study may broaden the spectrum of diseases, provide preliminary evidence for clinical practice and promote the education of microbiota medicine, which enriches the scientific explanations of microbiota medicine as a branch discipline in clinical medicine (Zhang et al. 2023).

Although Patient 5 received WMT and showed no clinical response, his stool culture converted to negative accompanied by relief of abdominal symptoms 20 days after the procedure. Unfortunately, only stool culture, rather than PCR, was performed for this patient. This observation might suggest that the patient might still benefit from WMT beyond our observation period. However, given the retrospective nature of this study, it remains uncertain whether PCR results would have been consistent with the negative stool culture finding.

Although the TASS of Patient 4 was zero, pathogen colonization remains a fundamental challenge in refractory infection (Frencken et al. 2018). Human‐associated microbes are naturally integrated to colonize in the natural orifices of humans, reflecting the law of host‐microbe mutualism (Woodworth et al. 2023). Meanwhile, long‐term colonization of pathogenic bacteria may pose a persistent challenge in clinical practice. Previous research shows that pathogenic colonization often preceded infection (Qin et al. 2020; Sun et al. 2021) and may even be associated with increased infection (Tacconelli et al. 2019; Le Guern et al. 2023). Limited critical infection and decolonization therapies are still challenging in clinical practice (Laxminarayan et al. 2020; Chanderraj et al. 2023). The gut microbiota has been reported to contribute to colonization resistance (Ducarmon et al. 2019), while certain bacterial components may also exacerbate disease severity (Lesniak et al. 2022). In our study, three patients' pathogenic evidence turned negative after WMT. These preliminary observations tentatively suggest WMT may serve as a potential clinical decolonization strategy. Previous reports have indicated that FMT may offer a promising approach to addressing this therapeutic gap by eradicating pathogenic bacteria in potential manners (Woodworth et al. 2023). However, it should be noted that only Patient 4 underwent stool PCR testing for confirmation, whereas Patients 3 and 5 were assessed only by stool culture after WMT. Ideally, all patients would have undergone stool PCR testing following WMT to confirm negativity; nevertheless, as this was a real‐world study, several factors—including clinical decisions at the participating hospital and financial constraints faced by patients' families—precluded the performance of PCR testing in every patient.

FMT plays a role in anti‐infection in the intestine in several manners. It has been reported to help restore commensal microbiota diversity and function from a state of dysbiosis (Liu et al. 2022), re‐establish colonization resistance (Yoon et al. 2019), and reduce antibiotic resistance genes in microbiomes (Jouhten et al. 2016; Millan et al. 2016). Colonization resistance is defined as the capability of healthy gut microbiota to resist pathogenic colonization (Alavi et al. 2020; Spragge et al. 2023). The direct mechanism for FMT strengthening the colonization resistance of gut microbiota might include the secretion of antimicrobial peptides and metabolites (Sun et al. 2018), and competition for nutrients and niches (Spragge et al. 2023; Woodworth et al. 2023). The indirect strategies mainly include strengthening the intestinal barrier and enhancing the immune system (Shah et al. 2021). The delivery routes for WMT in this study included mid‐gut, colonic transendoscopic enteral tube (TET) and enema. Colonic TET was reported as a novel, safe, and convenient colonic delivery way for repeated WMTs with a high degree of patients' satisfaction (Peng et al. 2016; Zhang, Long, et al. 2020). It is a new pathway to microbial therapy (Yu et al. 2026), colonic drainage, and host‐microbiota interaction research (Wang et al. 2023). In addition, it was recently reported to help patients with acute intestinal obstruction to avoid emergency surgery successfully (Yu et al. 2023).

The present study has several limitations. First, this was a single‐arm, uncontrolled research, which represents a fundamental methodological constraint. The absence of a concurrent or historical control group precludes definitive attribution of the observed clinical improvements to WMT, as spontaneous disease resolution, concurrent antimicrobial therapy, and supportive care may have contributed to the observed outcomes. Establishing a control arm (e.g., placebo or standard care) in this critically ill patient population with severe refractory intestinal infections is clinically challenging due to ethical concerns (withholding potentially life‐saving therapy), heterogeneity in disease presentation, and the urgent need for individualized treatment. Consequently, our findings should be interpreted as exploratory and hypothesis‐generating rather than confirmatory. Second, the small sample size limited further investigation into the exploration of mechanisms of WMT for decolonization and anti‐infection. Third, the heterogeneity in underlying diseases, pathogen types, and WMT delivery routes, while reflecting real‐world clinical practice, complicates the interpretation of efficacy signals. Finally, WMT rescue from CMTS is also distributed throughout the county, making specimen collection challenging.

Future research could use more rigorous yet feasible designs, such as a randomized pilot study or a propensity‐matched cohort study, to strengthen causal inference; until then, our findings suggest the potential utility of WMT. Besides, biotechnology could complement WMT via engineered bacteria for narrow‐spectrum antimicrobial activity to improve treatment precision (Álvarez and Fernández 2017; Brito et al. 2025). Future research should explore the potential synergy between WMT and engineered bacteria in the treatment of refractory infections (Timmis et al. 2019; Seco and Fernández 2022).

## 5 Conclusion

In conclusion, this article offers hypothesis‐generating evidence supporting the feasibility and potential effectiveness of WMT for refractory gut infections caused by identified pathogens beyond CDI in patients with complex comorbidities. Our findings also imply a potential role for WMT in pathogen decolonization, a hypothesis that necessitates further investigation through controlled studies.

## Author Contributions

**Rujun Ai:** conceptualization, investigation, funding acquisition, writing – original draft, methodology, visualization, writing – review and editing, project administration, resources, supervision, data curation. **Xia Wu:** supervision, writing – review and editing, writing – original draft, conceptualization, software, data curation. **You Yu:** conceptualization, supervision, data curation, software, visualization. **Yeqing Wang:** data curation, supervision, validation, investigation. **Quan Wen:** conceptualization, methodology, validation, visualization, writing – review and editing, writing – original draft, supervision. **Faming Zhang:** conceptualization, methodology, data curation, supervision, resources, funding acquisition, writing – review and editing, formal analysis, validation. **Yuyan Xiao:** supervision, resources, visualization, investigation. **Zulun Zhang:** conceptualization, validation, supervision, resources. **Zhihua Zhang:** investigation, validation, data curation, supervision. **Aoli Diao:** conceptualization, methodology, software, data curation, writing – review and editing, writing – original draft, investigation, resources, formal analysis, validation, visualization. **Xinyi He:** conceptualization, validation, project administration, supervision. **Bota Cui:** conceptualization, software, data curation, supervision, formal analysis, resources, writing – review and editing, writing – original draft, funding acquisition. **Jie Xu:** conceptualization, methodology, software, data curation, formal analysis, validation, investigation, funding acquisition, visualization, project administration, resources, writing – original draft, writing – review and editing, supervision.

## Funding

This work was supported by Natural Science Foundation of Jiangsu Province (BK20251961) and Jiangsu Provincial Health Commission (K2023011).

## Consent

The authors have nothing to report.

## Conflicts of Interest

Faming Zhang conceived the concept of GenFMTer and transendoscopic enteral tubing and the devices (FMT Medical, Nanjing, China) related to them. Faming Zhang is the editor of Microbial Biotechnology. Faming Zhang is the editor‐in‐chief of Microbiota Medicine Research.

## Supporting information


**Table S1:** WMT delivery routes and courses.

## Data Availability

The data that support the findings of this study are available on request from the corresponding author. The data are not publicly available due to privacy or ethical restrictions.
