# Supplementary material for: Feasibility and Safety of Washed Microbiota Transplantation as Salvage Therapy for Refractory Non‐CDI Gut Pathogens in Critically Ill Patients
Source: Microb Biotechnol. 2026 Aug 13;19(8):e70431. doi: 10.1111/1751-7915.70431 (PMC13473751; doi:10.1111/1751-7915.70431)
Supplement: Supplementary file 1 [file MBT2-19-e70431-s001.docx]

**Supplement Table 1. WMT delivery routes and courses.**

| ***Pt*** | ***Gender*** | ***Microbiological Pathogen (stool sample)*** | ***Delivery route*** | ***Course of treatment*** | ***Frequency of WMT*** | ***Negative pathogenic evidence confirmation (days after WMT)*** |
| --- | --- | --- | --- | --- | --- | --- |
| *1* | *male* | *Candida glabrata* | mid-gut | 1 | 3 | / |
| *2* | *male* | *Klebsiella pneumoniae* | mid-gut | 1 | 3 | / |
| *3* | *male* | *Salmonella typhimurium* | colonic TET | 1 | 2 | 34 |
| *4* | *male* | *Clostridium botulinum type B* | enema | 1 | 4 | 10 |
| *5* | *male* | *Pseudomonas aeruginosa* | mid-gut | 1 | 3 | 20 |
| *6* | *male* | *Klebsiella pneumoniae* | mid-gut | 1 | 1 | / |
| 7 | *male* | *Klebsiella oxytoca* | mid-gut | 1 | 3 | / |
| *8* | *female* | *Enterococcus* | enema | 1 | 6 | / |
| *9* | *male* | *Klebsiella pneumoniae* | mid-gut | 1 | 2 | / |
| *10* | *female* | *Candida albicans* | colonic TET | 1 | 2 | / |

Pt, patient; TET, transendoscopic enteral tubing. Pt1 had a positive stool culture on follow-up after treatment, which was most likely due to post-infection colonization. The remaining patients did not undergo repeated stool culture or PCR testing during the follow-up period.
